# Assessing ICD-11 Gaming Disorder in Adolescent Gamers: Development and Validation of the Gaming Disorder Scale for Adolescents (GADIS-A)

**DOI:** 10.3390/jcm9040993

**Published:** 2020-04-02

**Authors:** Kerstin Paschke, Maria Isabella Austermann, Rainer Thomasius

**Affiliations:** German Center for Addiction Research in Childhood and Adolescence (DZSKJ), University Medical Center Hamburg-Eppendorf (UKE), Martinistraße 52, 20246 Hamburg, Germany; ma.austermann@uke.de (M.I.A.); thomasius@uke.de (R.T.)

**Keywords:** GADIS-A, gaming disorder, ICD-11, adolescents, scale development and validation, screening instrument

## Abstract

Background: Adolescents affected by Gaming Disorder (GD) show substantial impairments in daily functioning. GD was included in the 11th revision of the International Classification of Diseases (ICD-11) as a new diagnosis coming into effect in January 2022. An instrument to screen for GD in adolescents has not yet been published and is urgently needed for scientific research and clinical practice. Methods: In the present study, the ICD-11-based Gaming Disorder Scale for Adolescents (GADIS-A) was developed by clinical experts and scientists. It was validated with 819 frequent gamers of 10 to 17 years and a respective caregiver in an online survey. Criterion validity was examined by assessing gaming behavior, emotional dysregulation, and academic performance. Item structure was investigated by factorial analyses. ROC- and Latent Profile Analyses were computed for differentiation between GD and Non-GD. Results: In line with the ICD-11 approach and accounting for cognitive-behavioral symptoms and negative consequences equally, GADIS-A items were best described by two factors. The new instrument showed excellent internal consistency, good criterion validity, and excellent discriminatory power. Conclusions: GADIS-A is the first successfully validated questionnaire to assess ICD-11 GD in adolescents. Thus, it can significantly contribute to reliably identify affected adolescents in clinical and research settings.

## 1. Introduction

The pathological use of digital games is a serious disorder associated with substantial psychosocial problems. Children and adolescents are especially prone to engage in pathological gaming, as their capabilities of cognitive control are still under development [1,2,3]. Sleep disorders, deterioration of school grades, and family conflicts, as well as emotional, behavioral, and cognitive problems, are more common among young pathological gamers than among non- and normal-gaming peers [4]. Moreover, emotional dysregulation (ED) as a hallmark of psychopathology is associated with pathological gaming behavior in children and adolescents [5,6]. Gaming could be identified to serve as a stress coping instrument in adolescent problem-gamers with avoidance-related emotion-focused strategies (e.g., denial, behavioral disengagement) [7,8]. At the same time, the quantity of digital online and offline games on offer for the adolescent target group is increasing. Approximately 500 games are uploaded in the smartphone App Stores per day [9,10,11] while the smartphone is the most frequently used device in 94% of German minors [12]. Strong attachment to games might be created, as a lot of games specifically address young people’s interests and incorporate intermittent reward systems [13,14]. Epidemiological studies on pathological gaming in adolescents conducted in Europe, Asia, and Australia revealed prevalence rates ranging from 1.2% to 5.9% [4]. However, findings might be difficult to interpret due to the heterogeneity of measuring instruments applied. Early questionnaires to assess pathological gaming were developed based on systematic reviews on pathological gaming or criteria of other addictive disorders [12]. Recent studies mainly employed scales measuring the diagnostic criteria for Internet Gaming Disorder (IGD) that was included as a “condition warranting more clinical research and experience” before possible consideration “as a formal disorder” in the Diagnostic and Statistical Manual of Mental Disorders 5 (DSM-5) in 2013 [4,15]. IGD comprises nine criteria referring to gaming on the internet, or on any electronic device: (1) preoccupation with gaming, (2) withdrawal when not playing, (3) tolerance, (4) unsuccessful attempts to reduce or stop gaming, (5) giving up other activities, (6) continuation of gaming despite problems, (7) deceiving or covering up gaming, (8) gaming to escape adverse moods, and (9) risking or losing relationships or career opportunities due of excessive gaming [16].

In May 2019, the Gaming Disorder (GD) was included in the 11th revision of the International Classification of Diseases (ICD-11). For GD to be diagnosed, the following criteria concerning the on-/off-line gaming behavior (“digital gaming” or “video gaming”) must be present for at least 12 months: (1) impaired control over gaming, (2) increasing priority given to gaming over other activities, and (3) continuation or escalation of gaming despite the occurrence of negative consequences [17]. This behavior is distinguished from non-pathological gaming by the resulting clinically significant distress or impairments in important areas of functioning [18]. The latter comprises personal (psychological and/or physical well-being), social, educational, work, and financial areas [19]. Besides the diverging number of DSM-5 and ICD-11 criteria, symptoms and resulting impairments are weighted differently in both classification systems. According to ICD-11, symptoms and disability aspects need to be present both for the diagnosis to be fulfilled. This underlines the different conceptualization and diagnostic approach of the ICD-11 compared to the DSM-5 [20]. In their recent review King and colleagues (2020) identified 32 IGD assessment tools available in the English language and employed in 320 studies [19]. Accordingly, the instruments differed regarding “(1) conceptual and practical considerations; (2) alignment with DSM-5 and ICD-11 criteria; (3) type and quantity of studies and samples; and (4) psychometric properties”. No single instrument was found to be clearly superior but 5 scales, including the 9-item Internet Gaming Disorder Scale (IGDS) by Lemmens et al. [21], showed the best psychometric properties. All tools mainly converged on the items measuring impaired control and functional impairment. The continued use despite harm was the most inconsistently addressed item by the scales reflecting different approaches on the continuum of normal to pathological gaming. Moreover, different samples were addressed by different scales. Regarding the coverage of ICD-11 GD criteria, the screening instruments diverged significantly. Therefore, they cannot be easily and comparably applied for GD diagnosis. 

Ko et al. (2019) and Jo et al. (2019) calculated GD prevalence by means of the corresponding IGD items they had queried in a semi-structured interview [22,23]. By this procedure, Ko et al. (2019) revealed that 63.8% of adults who met the criteria for IGD fulfilled the GD criteria of the ICD-11. Moreover, 25% of subjects with IGD were categorized as Hazardous Gamers (HG). According to ICD-11 HG, they show a gaming behavior that increases the risk of substantial negative consequences [17]. In line with these findings, Jo et al. (2019) showed that about one fifth of children and adolescents with DSM-5 IGDS met the ICD-11 GD criteria. As the ICD-11 seems to imply a higher diagnostic threshold than the DSM-5 [22,23], one could argue that incidence rates of pathological gaming might be underestimated by GD criteria. However, the IGD criteria were derived by the criteria for substance use disorders. Similar to substance use, fulfilling some addiction criteria might indicate extensive usage or a kind of normative behavior rather than pathology [24]. In line with this, Sugaya et al. (2019) revealed that some IGD criteria are more common in children and adolescents than others, which supports the suggestion of Colder Carras and Kardefelt-Winter (2018) to classify DSM-5 criteria in “weak” and “strong” symptoms of pathological gaming [4,24]. 

The first ICD-11 GD assessment tool was published by Pontes et al. [25]. They developed the Gaming Disorder Questionnaire (GDT), a brief standardized four-item assessment tool for adults reflecting the three GD symptom criteria and significant impairments associated with those. Using the GDT, the authors identified a comparably small proportion of 1.8% pathologic gamers in a sample of adult gamers from China and England.

A further difference between DSM-5 and ICD-11 pertains to temporal aspects of the pathological gaming: whereas DSM-5 refers to the occurrence of IGD symptoms within the last 12 months only, ICD-11 states that the pattern of gaming behavior may be continuous or episodic and recurrent, and normally evident over a period of at least 12 months. Besides, the required duration may be shortened to assign the diagnosis if all diagnostic requirements are met and symptoms are severe [17]. Consequently, in addition to ICD-11 symptoms and impairments, a GD-screening instrument should include time issues.

Since IGD and GD criteria cannot be compared directly, Pontes et al. emphasize the need for new standardized tools with good psychometric properties to assess GD according to the new diagnostic framework. As a further aim they suggest to investigate the relevance (and the weight) of each new criterion by cross-cultural clinical and psychometric research [25]. 

Although the ICD-11 will come into effect in January 2022, a diagnostic instrument to assess GD in children and adolescents is still missing. The gathered findings highlight the need to develop a questionnaire that assesses GD in children and adolescents, thereby enabling a distinction between pathological, hazardous, and normal gaming based on the ICD-11 division of symptoms, impairments and temporal aspects. Therefore, the aims of this study were (1) the development of a GD screening instrument for children and adolescents (Gaming Disorder Scale for Adolescence, GADIS-A), (2) the investigation of psychometric properties of the newly developed scale, and (3) the validation of GADIS-A in a sample of 10- to 17-year-old children and adolescents in Germany. To the best of our knowledge, no validated GD screening instrument for children and adolescents is available at this point. Hence, the present study has the potential to make an important contribution to the assessment of GD in this important young target group, thereby enabling standardized approaches for diagnostic screening in epidemiological, clinical, and neuroscientific research fields.

## 2. Materials and Methods

### 2.1. Participants and Procedure

A representative sample of 1221 German adolescents between 10 and 17 years and a respective caregiver participated in the current study. The procedure followed the ethical guidelines of the relevant national and institutional committees on human experimentation, was in accordance with the Declaration of Helsinki, and approved by the local ethics committee. Each adolescent and his/her caregiver gave their informed consent prior to participation. Data were collected by the German market research and opinion polling company Forsa via online survey. Participants were selected on the basis of all available representatives from a cluster of German adults aged 28 to 75 years. Respectively, more than 20,000 representative households were contacted via E-Mail (N = 23,736). Of N = 12,427 respondents, N = 1733 reported having children in the age of interest. A final number of N = 1221 provided necessary details and gave consent for participation. Among households with more than one child between 10 and 17 years, surveys were only conducted with the minor whose birth date was the most recent at the time of the survey to ensure a balanced proportion of age groups and gender. The adolescents completed the reported questionnaires as part of an online battery of psychometric instruments. This resulted in an overall average response time of 26 min including breaks. 

N = 1014 adolescents reported a regular use of digital games (at least once a week). This sample of gamers consisted of 40.1% girls (*n* = 407) and 59.9% boys (*n* = 607). In the sample, 92.9% (*n* = 947) adolescents attended school, 5.4% (*n* = 55) were trainees, and 1.7% (*n* = 17) were either university students, in voluntary service, military service, other occupation, or unemployed. The mean age was 13.01 (standard deviation [SD] = 2.36). Graduation level or prospective graduation level based on the current school performance revealed 54.7% high, 36.9% medium, and 8.4% low education.

### 2.2. Measures

#### 2.2.1. Gaming Disorder

GD symptoms according to the ICD-11 criteria were assessed by the newly developed Gaming Disorder Scale for Adolescence (GADIS-A). GADIS-A was created and consulted by clinical and scientific experts (specialist/consultants for child and adolescent psychiatry, scientists in the field of behavioral addictions). It comprises nine symptom statements with five (Likert-scale) response options plus one additional item regarding the frequency of symptoms with four response options. Table 1 shows the English GADIS-A version with the corresponding ICD-11 criteria (for the original German version refer to Table S1). For a better comparison, the corresponding DSM-5 items are also displayed. The participants were asked to indicate how strongly they agree with the given statements thinking of the last 12 months (strongly disagree—0, somewhat disagree—1, partially agree/ partially disagree—2, somewhat agree—3, strongly agree—4). Two items were formulated for the symptoms A to C leading to six items. Additionally, impairment (D) was addressed by three items reflecting personal, social, and educational/work aspects. By summing up the responses for the nine items, a total score was calculated leading to a possible maximum score of 36. The temporal issue (E) was considered by an item asking for the frequency of the mentioned problems, conflicts, or difficulties due to their gaming behavior within the last 12 months (answers: 0—not at all, 1—only on single days, 2—for longer periods, 3—nearly daily). A score of at least 2 was required to be reached to fulfill the time criterion. Content validity, feasibility, and comprehensibility were tested with adolescent patients with a clinically diagnosed gaming disorder treated in-patient and in day-clinic. 

To assess Internet Gaming symptoms according to DSM-5 the well-established Internet Gaming Disorder Scale (IGDS) was used in the internal- [26] and external-judgement version (PIGDS) [27]. The IGDS is a one-factor, polythetic instrument consisting of nine questions with a dichotomous response format (no/yes). It had been repeatedly applied to German adolescent samples and could proof good psychometric properties [19,28,29,30]. The parental version of the IGDS could be shown to be a valid and psychometric useful instrument to add external symptom views and had been validated in a sample of German adolescents and their caregivers [27]. A higher total sum of the yes-responses of both scales indicates a higher risk for IGD. The internal consistency for the sample of the current study was good (IGDS: Cronbach’s α = 0.83; PIGDS: Cronbach’s α = 0.84). Item 4, 6, 8, and 9 of the IGDS reflect ICD-11 associated criteria and were therefore considered separately to estimate the prevalence of ICD-11 symptoms [22,23].

#### 2.2.2. Gaming Pattern

Three questions were included in the survey measuring the average frequency of gaming per week (in days), per school day, and per weekend day (in hours). Out of the latter two, a mean gaming time was computed (in hours).

#### 2.2.3. Emotional Dysregulation

Emotional dysregulation could be revealed as a transdiagnostic instrument of mental health problems including substance and behavioral addictions [31,32,33]. It was assessed by the short form of the Difficulties in Emotional Regulation Scale (DERS-SF) [34]. This 18-item-scale is based on the 36-item DERS [35] and comprises the factors strategies (for emotional regulation), non-acceptance (of feelings), impulse (impulsiveness), goals (achievement), awareness (of feelings), and clarity (about feelings). Higher total and scale scores indicate higher emotional dysregulation. The instrument was validated in samples of German high school students [36]. Cronbach’s α of the current whole scale was 0.90 suggesting excellent internal consistency. Cronbach’s α of the subscales was 0.79 for strategies, 0.70 for non-acceptance, 0.90 for impulse, 0.83 for goals, 0.68 for awareness, and 0.81 for clarity. The scale awareness was excluded from subscale analysis due to its questionable consistency. 

#### 2.2.4. Academic Functioning

Academic functioning was defined by the rate of school or work attendance and academic performance. Attendance rates were measured by absence times from school/work during the last three months. Academic performance was assessed by the past-term final grades of the three main school subjects (German, Mathematics, first foreign language) each ranging from 1 (very good performance) to 6 (insufficient performance). The three grades were summed up to form global grade scores with higher scores indicating poorer academic performance. Moreover, performance was measured by the overall grade development during the last year (1—significant decline, 2—mild decline, 3—constant performance, 4—mild improvement, 5—significant improvement). 

### 2.3. Analysis

#### 2.3.1. Data Management and Analytic Strategies

Data from subjects with missing values of more than one third for each instrument (GADIS-A, IGDS, PIGDS, and DERS) were excluded from further analysis. Data of subjects that did not respond to the ICD-11 related timing question of the GADIS-A (on how often they experienced problems, conflicts, or difficulties due to their gaming behavior within the last 12 months) were also removed. This led to a total number of N = 195 excluded cases. Using the package mice of the statistical program R [37,38] multiple imputations were carried out regarding missing values of the remaining N = 819 subjects resulting in overall replacements per instrument of 0.2% (GADIS-A), 1% (IGDS), 2.6% (PGDQ), and 1.3% (DERS).

#### 2.3.2. Factor Structure

Following a split-half cross-validation method the sample was randomly divided in two (nearly) equal proportions using the R package rsample [39] (N_1_ = 410; N_2_ = 409). Subsequently, an exploratory factor analysis (EFA) was performed on the GADIS items of the first and a following confirmatory factor analysis (CFA) on the second subsample with the R packages psych and lavaan [40,41]. To affirm the suitability for factor analysis the Kaiser-Meyer-Olkin (KMO) criterion and Bartlett’s test of sphericity were determined on each subsample. Normality distribution was revised by determining skewness and kurtosis. Absolute values of skewness > 2.0 and kurtosis > 7.0 were used as reference values to determine substantial non-normality [42]. Since the GADIS-A comprises ordered categorical variables a robust minimal residuals (OLS) factoring was conducted [43]. For the first subsample, the Wayne Velicer’s Minimum Average Partial (MAP) criterion was used to determine the appropriate number of factors. The goodness of fit of the component structure tested with CFA was evaluated by the chi-square value, the root mean square error of approximation (RMSEA), the standardized root mean squared residual (SRMR), the Tucker-Lewis Index (TLI). Moreover, the comparative fit (CFI) was calculated. Model goodness of fit was assumed according to the following criteria: RMSEA < 0.1 [44], SRMR < 0.08, TLI ≥ 0.95 [45], CFI ≥ 0.95 [46]. According to the best factorial solution, sum scores were computed for each factor scale.

#### 2.3.3. Internal Consistency

Internal consistency was determined using Conbach’s α and McDonald’s ω with coefficients ≥ 0.70 being regarded as acceptable [47].

#### 2.3.4. Criterion Validity

Criterion validity was obtained by correlating the GADIS-sum score with the IGDS- and PIGDS-sum score, the gaming days per week, as well as the mean gaming hours per day using correlation tests. Moreover, correlations were calculated for the GADIS-sum score and the DERS-SF-sum score including the subscales impulse, strategies, non-acceptance, goals, and clarity. To address academic functioning, correlations were calculated for the GADIS score and days of absence (during the last three months), grades-sum score, and grade development. Pearson or Spearman rank correlations were computed according to the item/scale distribution. Absolute correlation coefficients were interpreted as follows: Pearson: *r* ≥ 0.70 strong relationship, 0.40 > *r* ≥ 0.69 moderate relationship, 0.10 > *r* ≥ 0.39 weak relationship, 0.01 > *r* ≥ 0.09 no relationship; Spearman: ϱ ≥ 0.70 very strong relationship, 0.40 > ϱ ≥ 0.69 strong relationship, 0.30 > ϱ ≥ 0.39 moderate relationship, 0.20 > ϱ ≥ 0.29 weak relationship, 0.01 > ϱ ≥ 0.19 no or negligible relationship [48].

#### 2.3.5. Classification

Classification was realized by a latent-profile analysis (LPA) on the GADIS-factor sum scores and the frequency of gaming-associated problems/impairments using the R packages mclust and tidyLPA [49,50]. LPA provided an estimate of the number of latent subgroups regarding gaming disorder within the sample and the number of adolescents in each subgroup. The ideal number of profiles was determined by the bootstrap likelihood ration test (BLRT), the Akaike information criterion (AIC), the Bayesian information criterion (BIC), and the Integrated Completed Likelihood (ICL). The BLRT uses bootstrap samples to estimate the distribution of the log likelihood difference test statistic. It contrasts the fit between a certain model and a model with one profile less [51]. The null hypothesis states that the smaller model is the best model. It is rejected when a statistically significant better fit of the larger model is indicated by *p* < 0.001. Additionally, lower values of BIC, AIC, and ICL reflect better model solutions [52,53]. All profile groups were described regarding prevalence and sex, using frequency calculations with 95% confidence intervals (CI), as well as age, GADIS-A-, IGDS-, PIGDS-, and DERS-SF-sum scores, gaming days per week, gaming hours per day, absence, grade-sum score, and grades development using means with standard error of means (se). All (dependent) variables were included in a MANOVA with the latent profile group as independent factor. Significant results were further evaluated using post-hoc χ^2^– and Scheffé tests with Bonferroni-corrected *p* values. Effect sizes were computed using Cramer’s V (categorial variables) and Cohen’s d with the following interpretation of the absolute values: Cramer’s V > 0.05 weak, > 0.1 moderate, > 0.15 strong, > 0.25 very strong effect; Cohen’s d > 0.2 small, > 0.5 medium, > 0.8 large effect [54].

#### 2.3.6. Sensitivity and Specificity

A receiver operating characteristic curve (ROC) analysis was conducted to compare sensitivity and specificity across GADIS-A- scores to predict Gaming Disorder according to ICD-11 related IGDS items using the R package pROC [55]. In this analysis, 999 bootstrapping replications were applied to define 95%-confidence intervals. A cut-off point was determined based on Youdon’s criterion. Goodness of differentiation between the two diagnostic groups was ascertained by the area under curve value (AUV) with values above 90% indicating excellent differentiation. The received cut-off point was then applied to classify adolescents with GD if they started to experience gaming associated problems over longer periods or daily within the last 12 months (accounting for the ICD-11-time criterion). Besides estimating prevalence, the two groups based on the GADIS-cut-off point were compared regarding age, sex, GADIS score, IGDS and PIGDS score, gaming days per week, gaming hours per day, absence, grade-sum score, and grades development by a MANOVA with post-hoc χ^2^ and Scheffé tests. Moreover, effect sizes were calculated (Cramer’s V and Cohen’s d, for interpretation see Section 2.3.5).

## 3. Results

### 3.1. Factor Structure

Bartlett’s test revealed significant correlations between the nine GADIS-A items on both halves of the sample data (χ2(36) = 2395.49, *p* < 0.001 and χ2(36) = 2420.96, *p* < 0.001). KMO criterion was 0.89 and 0.9 overall for both sub-samples and ranged between 0.85 and 0.96 for individual items. Thus, an excellent suitability of the data for factorial analyses could be demonstrated.

EFA computed on half of the sample strongly suggested a 2-factor solution (eigenvalue factor 1 = 5.26; eigenvalue factor 2 = 1.3; maximum VSS complexity of 0.96; minimum Velicer MAP of 0.06; minimum empirical BIC of −71.21). Communalities ranged from 0.58 to 0.74. The cumulative variance explained by the two factors was 0.65 (variance of factor 1 = 0.35). Factor loadings ranged between 0.63 and 0.84 for factor 1 and 0.6 and 0.82 for factor 2.

**Figure 1:**
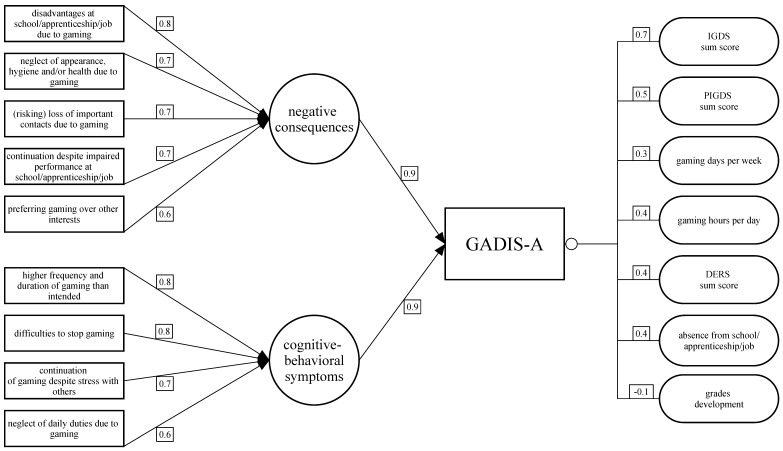
Factor loadings on two GADIS-A factors. GADIS-A = Gaming Disorder Scale for Adolescents, GADIS-A factor 1 = negative consequences due to gaming. Items loading on factor 1 as presented on the left side: 7-9, 6,3; GADIS-A factor 2 = cognitive-behavioral gaming symptoms. Items loading on factor 2 as presented on the left side: 1,2,4,5; IGDS = Internet Gaming Disorder Scale, PIGDS = Parental Internet Gaming Disorder Scale, DERS = Difficulties in Emotion Regulation Scale, Grades development = improvement of the sum of grades of the three main subjects during the past school year.

Applying the 2-factor solution to CFA revealed an overall good fit to the data (standard χ2(26) = 123.09, *p* < 0.0001, robust χ2(26) = 232.65, *p* < 0.0001, RMSEA = 0.096, SRMR = 0.057, CFI = 0.995, TLI = 0.993). GADIS-A items 7 to 9 (personal, social, and academic/occupational impairments), 6 (continuation despite academic/occupational disadvantages), and 3 (loss of other interests due to gaming), loaded highest on factor 1, thus, reflecting impending or manifest consequences due to gaming. Additionally, GADIS-A items 1 and 2 (loss of control), 5 (continuation despite social stress) and 4 (neglecting daily duties) loaded highest on factor 2. Therefore, factor 2 can be best described as mirroring cognitive-behavioral gaming symptoms. EFA- and CFA-factor loadings are shown in Figure 1, and additional EFA communalities and variance proportions, are shown in Table 2. Tables S2 and S3 present inter-item correlations and the relative item-response frequencies. All items showed a moderate correlation with the additional timing question (0.42 ≤ *r* ≤ 0.68).

### 3.2. Internal Consistency

For the total GADIS-A scale a Cronbach’s α of 0.91 and a McDonald’s ω of 0.94 was calculated reflecting excellent internal consistency. For the subscale regarding the first factor Cronbach’s α revealed a value 0.9 and McDonald’s ω of 0.92. For the second factor subscale a Cronbach’s α of 0.87 and a McDonald’s ω of 0.9 was computed. Thus, the total GADIS-A and the first subscale reflect excellent and the second subscale good internal consistency.

### 3.3. Criterion Validity

Skewness and kurtosis of all items and scales revealed no substantial deviance from normal distribution except for the mean gaming time per day and the days of absence. Analyses revealed strong positive correlations between the GADIS-A-sum score and the amount of fulfilled DSM-5 criteria of problematic gaming (gathered by IGDS sum score, *r* = 0.7, *p* < 0.001) indicating excellent criterion validity. Moderate positive correlations could be revealed between GADIS-sum score and the number of fulfilled DSM-5 criteria according to the parental assessment via PIGDS (*r* = 0.54, *p* < 0.001), gaming days per week (Spearman’s ϱ = 0.3, *p* < 0.001), and the mean gaming hours per day (*r* = 0.42, *p* < 0.001). Moreover, the DERS-sum score as a measure of emotional dysregulation correlated positively with the GADIS-A-sum score in a moderate manner (*r* = 0.51, *p* < 0.001, subscales *r* = 0.38 to 0.41, *p* > 0.001). These results reflect an overall good criterion validity. Weak positive correlations were found for the GADIS score and the days of absence from school or work (Spearman’s ϱ = 0.2, *p* < 0.001). The grades development correlated significantly negative with the GADIS score although the correlation was small as well (*r* = −0.11). A significant but negligible positive correlation was calculated for the GADIS-sum score and the sum of the grades of the three major subjects at school (*r* = 0.09). The correlation values and their significance levels are shown in Table 3 and are visualized in Figure 1.

### 3.4. Classification

Based on the latent profile analysis including the sum of the two GADIS-subscale scores and the frequency of impairments an ellipsoidal, equal volume, and shape model with four profiles showed the best fit with smallest AIC, BIC, and ICL values (see Table 4). According to the LRT a two- or a four-profile model would be appropriate. However, considering both requirements of a small number of profiles and a good fit, the four-profile solution is clearly favored. Pillai score (df = 3815) of the MANOVA concerning the four profile groups and 13 dependent variables was 1.28 with (F(39,2415) = 46.38, *p* < 0.001). It revealed significant differences between the groups regarding the variables sex, GADIS-A sums of factors 1 and 2, frequency of GADIS-A symptoms, IGDS, PIGDS, and DERS sum score, gaming frequency and duration, days of school absence, and academic performance development. No significant differences were found for age and the sum of the three main school subjects. F values and *p* scores of each variable can be found in Table S4.

According to the GADIS-A profile and the time spent with gaming, group 1 could be referred to as hazardous gamers (HG), group 2 as intensive gamers (IG), group 3 as pathological gamers (PG according to GD), and group 4 as light gamers (LG). LG reported no prolonged problems (100% [CI 100; 100]) and IG stated problems on single days during the last year (99.79% [99.39; 100.02]). In contrast, HG reported problems for longer periods (56.25% [31.94;80.56]) or even daily (43.75% [19.44;68.06]). Comparable to HG, PG stated problems to be present over longer periods of time (52.17 [37.74;66.61]) or daily during the last year (47.83% [33.39;66.26]). IG and LG comprised almost all subjects (N_IG_ = 481, 58.7% and N_LG_ = 276, 33.7%) whereas the remaining two groups included N_HG_ = 16 (2%) and N_PG_ = 46 (5.6%) adolescents. IG and LG did not differ regarding days of absence and grades development. Hence, compared to LG, IG was comprised of less girls (34.79 vs. 52.17%), received higher gaming behavior associated scores (GADIS subscale 1 and 2, IGDS, PIGDS, gaming frequency and duration), and slightly higher DERS sum scores (33.84 vs. 26.42) with medium to (very) large effect sizes. HG did not differ from PG according to sex, frequency of GD symptoms, IGDS and PIGDS sum score, gaming frequency and duration, DERS sum score, and the grades sum indicating a high-risk profile of HG. Strikingly, HG and PG could be clearly differentiated by both GADIS-A subscales: PG reached significantly higher factor 1 and factor 2 sum scores with very large effect sizes (d_1_ = 2.12, d_2_ = 1.23). Whereas PG exceeded both subscale cut-offs, HG were above threshold only on the cognitive-behavioral symptoms scale. They did not reach the cut-off regarding negative consequences due to their gaming behavior. This finding is in line with a worse grades development of PG compared to HG over the past year on a non-corrected significance level with a medium (close to large) effect size. Please refer to Table 5 for more details on means and relative frequencies, standard errors of means (SE) and confidence intervals, post-hoc comparison values, and effect sizes.

### 3.5. Sensitivity and Specificity

According to Ko et al. [22], we chose the ICD-11 equivalent items of the DSM-5 criteria measured by IGDS to divide the sample in adolescents with and without Gaming Disorder. This classification was included into three ROC analyses together with the global GADIS and the sum scores of the two factors to address the two-factor structure of the ICD-11-derived scale. Applying Youden’s criterion the optimal cut-off for the global score was 12.5 (CI 12.5; 15.5) with a specificity of 83.12% (CI 79.61; 91.31) and a sensitivity of 97.96% [CI 89.8;100]. An AUC value of 95.1% [CI 92.7; 97.4] indicated an excellent differentiation between the two diagnostic groups by the cut-off. For factor 1, a cut-off value of 5.5 (CI 2.5; 6.5) with a specificity of 86.62% (CI 69.21; 92.21) and a sensitivity of 85.71% (CI 73.47;97.96) was computed. AUC value was 91.7% (CI 88.2; 95.2%). Factor 2 was associated with a cut-off value of 9.5 (CI 7.5;9.5) with a specificity of 85.45% (CI 77.14, 89.87%), sensitivity of 93.88% (CI 83.67; 100), and an AUC value of 93.8% (91.0; 96.6%).

Considering the ICD-11-time item (symptoms at least for longer periods or daily) and applying the cut-off of > 5 for factor 1, > 9 for factor 2, and ≥ 12 for the whole scale, 3.7% [CI 2.4; 5.0] of the adolescent gamers were classified as gaming disordered (GD, N = 30). Except for age, sex, and grades-sum score, all dependent variables (GADIS-A-subscale sum scores, frequency of GD symptoms, PGDQ-, and IGDS-sum score, days of gaming per week, mean gaming hours per day, DERS sum, absence, grades sum and grades development) reached significance when being included in a MANOVA with the factor gaming disorder (yes/no) (Pillai score (1817) = 0.43, F(13,805) = 20.6, *p* < 0.001; Table S4). Whereas 36.67% [19.42; 53.91] of GD adolescents reported problems during longer periods, 63.33% [46.09; 80.58] of this group stated symptoms to be present almost daily. In contrast, 34.98% [31.65; 38.31] of Non-GD adolescents reported no prolonged problems and the remaining 60.84% [57.43; 64.24] problems on single days only. Strong effects were revealed with higher GADIS-, IGDS-, PGDQ-, and DERS-sum scores as well as a longer gaming duration per day in GD adolescents compared to the majority of gamers. Moderate effects could be shown for the affected gamers regarding a higher gaming frequency per week and more days of absence from school or work. The academic achievement was worse in the GD compared to the unaffected group with small effect sizes. Moreover, on a descriptive level the percentage of girls was smaller in the affected adolescents (26.7% vs. 40.7%) although no significance was reached. Frequencies and confidence intervals, means and standard error of means, as well as post-hoc test and *p* values, are shown in Table 6.

## 4. Discussion

The Gaming Disorder Scale for adolescence (GADIS-A) is a newly developed instrument to assess the ICD-11 criteria of Gaming Disorder (GD) in children and adolescents. It comprises nine GD-symptom items plus 1 item reflecting the frequency of GD symptoms according to the ICD-11-time criterion. Our aim was to provide a valid and reliable diagnostic-support instrument. To the best of our knowledge, no alternative ICD-11-based questionnaire for adolescents is available thus far. Yet, a brief and easy screening tool administrable in busy clinical settings or for scientific surveys is needed to detect and better understand potentially pathological gaming behavior.

The GADIS-A could be shown to be psychometrically robust and highly feasible at the same time. Factor analyses favor a two-factor solution to best model the nine GADIS-A items. The first factor mirrors impending or manifest consequences due to the gaming behavior which will become significantly apparent after a longer time. It comprises impairments in psychosocial functioning and is related to the Disability Diagnostic Scale (DDS) and the Global Assessment of Functioning (GAF) according to ICD-10 and DSM-IV. The corresponding multiaxial system for mental diseases is a standard diagnostic approach in the clinical practice of psychiatry [56,57]. Contrary to the ICD-10 approach, this aspect is now explicitly included in the ICD-11 definition of mental disorders and helps in evaluating gaming behavior on a continuum between mental health and illness [18]. Pathological gaming behavior is best described under the second GADIS-A factor which includes immediately observable cognitive-behavioral symptoms such as prolonged gaming times, the inability to stop gaming, or the neglect of daily duties. The items of both factors are not completely independent which is mirrored by weak to moderate cross factorial inter-item correlations. This might explain why GADIS-A item 5 (continuation despite social stress) loads higher on the cognitive-behavioral symptoms than the consequences factor. Moreover, whereas the factor 1 consequences will become apparent over time, social stress is usually a consequence in the immediate aftermath of pathological behavior. Patients and parents commonly report the development of a withdrawal from the child-parents interaction to escape potential fights. Conversely, GADIS-A item 3 (loss of other interests due to gaming) could also be interpreted as a personal/social consequence that will become apparent over time. The two-factor solution underlines the new symptom weighting by the ICD-11 compared to the DSM-5. In the latter functional impairments are reflected by two out of nine symptoms all loading high on one IGDS factor [19,21]. Since every item is weighted equally, an IGD diagnosis can be obtained without an impairment symptom at all. By the two-factor ICD-11 approach psychosocial consequences and gaming specific cognitive-behavioral symptoms are weighed equally. In this way, the significance of clinical symptoms can be derived more clearly, thus, enabling the development and application of treatment and intervention strategies. Moreover, the 2-factorial GADIS-A structure is in line with the biaxial model of addiction. Accordingly, to define an addictive behavior as disordered both specific symptoms and adverse outcomes must occur [58]. Colder Carras and Kardefelt-Winter [24] argue for a careful consideration of addiction-related symptoms and gaming-related problems at the same time. In their large-scale investigation of 7865 adolescent European gamers only 2.2% showed both symptoms and problems. Beyond that, 30.9% of the sample would have been misclassified according to their reported personal problems. 7.3% of engaged gamers reported many symptoms but only a few problems, whereas 23.6% of concerned gamers indicated only a few symptoms but a high level of gaming-associated problems. Although Pontes et al. (2019) state that GD is best conceptualized within a single-factor structure based on their four-item GDT [24], the biaxial model would clearly simplify distinguishing pathological gamers from (i) hazardous gamers who meet symptom criteria but do not experience substantial impairments, (ii) non-hazardous gamers who fulfill none or only sub-threshold levels of symptoms and/or impairments, as well as from (iii) gamers with insignificant symptoms but substantial impairments that may rather be associated with behaviors other than gaming. As a result, the risk of classifying gamers who play extensively but do not experience substantial impairments as pathological might be reduced [23]. Hence, subjects will only be labeled with GD when fulfilling both criteria: cognitive-behavioral symptoms and negative consequences resulting from these symptoms. Support comes from the newly developed Digital Addiction Scale for Children (DASC): Hawi et al. revealed the presence of two factors underlying their 25-item questionnaire on digital-media addiction by an exploratory factor analysis [59]. Whereas factor 1 included conflict, displacement, and problems criteria, factor 2 reflected mood modification, withdrawal, and tolerance criteria.

Another indication for the meaningfulness of a differentiation between negative consequences and cognitive-behavioral gaming symptoms comes from the high rate of spontaneous remission of media-addiction symptoms—up to 76% within one year [60,61,62]. Spontaneous remission will be more likely when impairments due to gaming are not present or not severe. This might be the case for hazardous gamers according to ICD-11.

Moreover, a risk of GD overestimation will become more evident when including smartphone gamers besides computer and console gamers. According to Hawi et al. 12.4% of children aged 9 to 12 years are addicted to their digital devices (tablets, smartphones, and game consoles) used for gaming, social media, texting according to the 9 DSM-5 criteria [59]. For example, 30.9% of middle school students in South Korea [63] and 16.9% of Swiss adolescents were classified as smartphone addicted [64]. Those findings highlight the need for diagnostic instruments on GD that measures symptoms of clinical and temporal significance and reach both high sensitivity and high specificity.

Thus, when assessing one-year symptoms in younger and older adolescents possible recall biases should be considered [65,66,67]. An overestimation of retrospective media use could be shown for adolescents [68]. Moreover, children and adolescents overestimate symptoms when filling out retrospective questionnaire compared to prospective diary [69]. Accordingly, judging symptoms over a one-year period might be a difficult task facilitating overestimation. To account for that, an additional question regarding the frequency of GD symptoms was included in the GADIS-A. This might help to retrieve more realistic memory information in adolescents.

Adding the ICD-11-time criterion (frequency of GD symptoms within the last 12 months), about 4% of the adolescents were classified as GD. They differed significantly from the rest of the sample regarding a lower percentage of girls, a larger amount of fulfilled IGD-DSM-5 criteria, longer and more frequent gaming times, higher rates of emotional dysregulation, and lower academic achievements. Considering confidence intervals, the prevalence estimation was comparable to the GD-prevalence estimation revealed by LPA. Thus, GADIS-A proofed excellent discriminatory power. The LPA-GD group showed above-threshold values for the GADIS-A-total and sub scales. Moreover, LPA GD scored highest on the same validation scales as ROC GD indicating an urge for appropriate treatment. The majority of gamers was classified as non-pathological (light [33.7%] and intensive [58.7%]) gamers by LPA. Their scores on the sub- and total scales did not reach the cut-off values retrieved by the performed ROC analysis. They reported GD symptoms during the last year not at all or on single days only. On the contrary, a small group (2.0%) of hazardous gamers (HG) reached the cut-off value of GADIS-A factor 2 but not factor 1. This indicated the presence of pathological cognitive-behavioral gaming symptoms but no severe impairments at this stage. HG differed from pathological gamers (PG) by underneath-threshold negative consequences and above-threshold but significantly lower cognitive-behavioral symptoms as well as marginally better development of grades. Strikingly, both groups scored similarly high on all other variables including the number of positive DSM-5 criteria, gaming frequency and duration, as well as emotional dysregulation supporting a high-risk profile of HG and the need for an early intervention.

Interestingly, new hypotheses on different etiologies of GD/HG could be derived and tested based on the two-factorial distinctions. The current study validated the GADIS-A with a nationwide representative sample of frequent gamers. To obtain more information about possible sub-groups of HG a sample with over-representative intensive gamers should be drawn. Desirably, future studies should focus on different profiles of hazardous gamers since different profiles will need to be adapted for (early) intervention approaches to enable an optimal recover. Besides, the following limitations of the current study should be considered: first of all, an online survey can only include people with Internet access. Although, 94% of the German population has home Internet access [70], a small proportion might not have been considered. Though, it is unlikely that adolescents without home Internet access are frequent gamers. Furthermore, using online questionnaires in epidemiologic surveys is highly common for economic reasons. Another limitation concerns the current sample of minors: the parents of all adolescents had to confirm participation. Thus, only potentially interested parents enabled a participation of their children. Despite respondents being asked to answer all items on their own, it also cannot be excluded that parents or other people present influenced their answers. In addition, social desirability needs to be taken into account leading to potential underestimations of problematic behavior. In contrast, an online-survey setting could lead to overestimations due its anonymous nature. As very young adolescents participated, it could also be questioned whether their introspective abilities were sufficient to judge all items properly. Though, the use of questionnaires in later childhood and adolescence was repeatedly shown to be valuable [71,72]. Furthermore, we were able to add the view of the parents on addictive symptoms. It correlated moderately positive with the adolescents’ answers. Nonetheless, highest accuracy would be obtained using clinical interviews in addition to information from parents or other close family members. The present representative cross-sectional survey is a generally suitable tool to estimate prevalence. Yet, no conclusions can be drawn about potential cause and effects of psychosocial features such as emotional dysregulation and absence from school. A further shortcoming is the missing criterion validity with objective markers such as logged game usage times. By follow-back questions a reporting bias must always be considered. Since the number of items in an online survey is restricted, not all potentially interesting psychosocial factors could be captured. In addition, as typical for large surveys, the current dataset was accompanied by missing values and subjects that had to be excluded due to incomplete response patterns. This might have reduced the representativeness but is thus a common cost of an economic research instrument (especially when investigating young adolescents). Another limitation might concern the computation of cut-off values for GD-/Non-GD differentiation. Defining cut-off values is based on a categorical approach, thereby neglecting a smooth transition from normal to problematic and pathological gaming. Cut-off values can nevertheless be helpful in clinical practice to detect GD at an early stage to prevent secondary diseases and chronifications. Finally, note that GADIS-A is not an instrument that can be solely relied on for GD diagnosis. It cannot replace clinical expertise but can rather function as an economic screening tool. Clinical validation in future studies is desirable to enable a broad usage in clinical settings.

## 5. Conclusions

GADIS-A is the first available screening instrument for the assessment of GD in children and adolescents according to ICD-11 with excellent internal consistency reliability and good criterion validity. It excellently differentiated between pathological and non-pathological gamers and could reveal a group of hazardous gamers. Moreover, its two-factorial structure supports the ICD-11 approach of concerning both gaming-related cognitive-behavioral symptoms and their negative consequences. Furthermore, by considering the time aspect of pathological gaming behavior, occasional problems can be distinguished from outlasting problems necessary for the diagnosis to be fulfilled. Thus, GADIS-A contributes to a highly sensitive and specific identification of pathological adolescent gamers essential for the choice of adequate treatment and valuable to research further.

## Figures and Tables

**Table 1:** GADIS-A items with corresponding ICD-11 and DSM-5 criteria.

ICD-11 Criteria		GADIS-A Items	
Corresponding DSM-5 Item		Thinking of the Last 12 Months, How Strongly Do You Agree with the Following Statements?	
A	Impaired control over gaming (e.g., onset, frequency, intensity, duration, termination, context).	1.	I often play games more frequently and longer than I planned to or agreed upon with my parents. ^1^
Unsuccessful attempts to reduce or stop gaming.		2.	I often cannot stop gaming even though it would be sensible to do so or for example my parents have told me to stop. ^1^
B	Increasing priority given to gaming to the extent that gaming takes precedence over other life interests and daily activities.	3.	I often do not pursue interests outside the digital world (e.g., meeting friends or partner in real life, attending sports clubs/societies, reading books, making music) because I prefer gaming. ^1^
Giving up other activities.		4.	I neglect daily duties (e.g., grocery shopping, cleaning, tidying up after myself, tidying my room, obligations for school/apprenticeship/job) because I prefer gaming. ^1^
C	Continuation or escalation of gaming despite the occurrence of negative consequences.	5.	I often continue gaming even though it causes me stress with others (e.g., my parents, siblings, friends, partner, teachers). ^1^
Continuation of gaming despite problems.		6.	I continue gaming although it harms my performance at school/apprenticeship/job (e.g., by being late, not participating in class, neglecting homework, worse grades). ^1^
D	The behavior pattern is of sufficient severity to result in significant impairment in personal, family, social, educational, occupational or other important areas of functioning.	7.	Due to gaming, I neglect my appearance, my personal hygiene, and/or my health (e.g., sleep, nutrition, exercise). ^1^
		8.	Due to gaming, I risk losing important relationships (friends, family, partner) or have lost them already. ^1^
Risking or losing relationships or career opportunities due to excessive gaming.		9.	Due to gaming I have disadvantages at school/apprenticeship/job (e.g., bad [final] grades, inability to continue to the next grade/no graduation, no apprenticeship or university spot, poor reference, warning/dismissal). ^1^
E	The pattern of gaming behavior may be continuous or episodic and recurrent and normally evident over a period of at least 12 months.	10.	How often did you experience such problems, conflicts, or difficulties due to gaming during the past year? Did this only occur on single days, during longer periods of several days to weeks, or was it almost daily? ^2^

GADIS-A = Gaming Disorder Scale for Adolescents, ICD-11 = 11th revision of the International Classification of Diseases, GD = Gaming Disorder, DSM-5 = 5th revision of the Diagnostic and Statistical Manual of Mental Disorders, IGD = Internet Gaming Disorder, ^1^ response options: 5-point Likert-Scale: “strongly disagree”- “strongly agree”; ^2^ response options: “not at all”, “only on single days”, “during longer periods”, “almost daily”.

**Table 2:** Exploratory Factor Analysis (EFA) and Confirmatory Factor Analysis (CFA), factor loadings, EFA communalities, and EFA explained variance proportions.

GADIS-A Item ^a^	Factor 1	Factor 2	Communalities
Item 1 EFA	0.17	0.82	0.70
Item 1 CFA		0.93	
Item 2 EFA	0.22	0.80	0.69
Item 2 CFA		1.00	
Item 3 EFA	0.63	0.47	0.61
Item 3 CFA	0.92		
Item 4 EFA	0.47	0.60	0.58
Item 4 CFA		0.94	
Item 5 EFA	0.39	0.74	0.69
Item 5 CFA		1.02	
Item 6 EFA	0.72	0.40	0.68
Item 6 CFA	0.98		
Item 7 EFA	0.75	0.20	0.60
Item 7 CFA	0.95		
Item 8 EFA	0.73	0.24	0.59
Item 8 CFA	1.00		
Item 9 EFA	0.84	0.21	0.74
Item 9 CFA	1.00		
Proportion Variance	0.35	0.30	

GADIS-A = Gaming Disorder Scale for Adolescents, EFA = Explanatory Factor Analysis, CFA = Confirmatory Factor Analysis. ^a^ For the description of the items, refer to Table 1. GADIS-A factor 1 = negative consequences, GADIS-A factor 2 = cognitive-behavioral symptoms, EFA by principal component analyses.

**Table 3:** Criterion validity—Pearson/Spearman correlations of GADIS-A sum score.

Construct	r/ϱ
IGDS sum score	0.70 ***
PIGDS sum score	0.54 ***
Gaming days per week	0.28 ***
Gaming hours per day	0.43 ***
DERS sum score (and subscale scores)	0.50 *** (0.38–0.41 ***)
Days of absence	0.20 ***
Grade sum score	0.08 *
Grade development	−0.15 ***

*** *p* ≤ 0.001, * *p* ≤ 0.05; GADIS-A = Gaming Disorder Scale for Adolescents, *r* = Pearsons correlation coefficient, ϱ = Spearmans correlation coefficient, IGDS = Internet Gaming Disorder Scale, PIGDS = Parental Internet Gaming Disorder Scale, DERS = Difficulties in Emotion Regulation Scale, days of absence in school/ at work/job, grade sum = cumulated grades of the three main subjects with higher scores indicating poorer performance, grade development = improvement of grades sum over last term.

**Table 4:** Comparison of number of latent classes according to Latent Profile Analysis (LPA).

Latent Classes	Log Likelihood	AIC	BIC	ICL	LRTS
1	−2921.85	5861.69	−5904.07	−5904.07	-
2	−2924.51	5881.03	−5956.36	−5975.72	−5.33
3	−2052.60	4151.19	−4259.48	−4259.48	1743.84 ***
4	−1892.55	3845.10	−3986.34	−3986.75	320.09 ***
5	−1892.55	3859.10	−4033.30	−4371.93	0.00

LPA = Latent Profile Analysis, AIC = Akaike Information Criterion, BIC = Bayesian Information Criterion, ICL = Integrated Completed Likelihood, LRTS = Likelihood Ratio Test Score based on bootstrapping with 999 replications, LRTS *p*-values = *** *p* ≤ 0.001.

**Table 5:** Post-hoc MANOVA and between groups tests of gaming profiles according to Latent Profile Analysis (LPA).

Variables	Hazardous Gamers (HG)	Intensive Gamers (IG)	Pathological Gamers (PG)	Light Gamers (LG)	Post-Hoc Tests (χ2/Scheffé) ^a^	Cramér’s V/Cohen’s d
**Absolute frequency**	16	481	46	276	---	---
**Relative frequency in % [95%-CI]**	1.95 [1.0; 2.9]	58.73 [55.4; 62.1]	5.62 [4.0; 7.2]	33.7 [30.5; 36.9]	---	---
**Age mean (SE)**	11.81 (0.55)	12.98 (0.11)	12.91 (0.28)	13.10 (0.15)	---	---
**Female sex in % [95%-CI]**	25.00 [3.8;46.2]	34.72 [30.5;39.0]	30.43 [17.14;43.7]	52.17 [46.3;58.1]	0.29 n.s.	---
					0.01 n.s.	---
					n.s.	---
					0.18 n.s.	---
					21.36 ***	0.17
					6.61 p_uncorr_ = 0.01	0.15
**GADIS-A factor 1 score** **mean (SE)**	1.50 (0.38)	2.90 (0.15)	11.00 (0.11)	0.55 (0.08)	1.40 n.s.	---
					9.50***	2.12
					−0.95 n.s.	---
					8.10 ***	2.27
					−2.35 ***	0.84
					−10.45 ***	4.67
**GADIS-A factor 2 score** **mean (SE)**	8.19 (0.84)	6.79 (0.14)	11.98 (0.44)	1.30 (0.08)	−1.40 n.s.	---
					3.79 ***	1.23
					−6.89 ***	4.62
					5.19 ***	1.72
					−5.49 ***	2.16
					−0.68 ***	6.48
**Frequency of GD symptoms score** **mean (SE)**	2.44 (0.13)	1.00 (0.00)	2.48 (0.07)	0.00 (0.00)	−1.43 ***	14.38
					0.04	---
					−2.44 ***	20.92
					1.48 ***	9.58
					−1.00 ***	27.56
					−2.48 ***	13.09
**IGDS sum score** **mean (SE)**	5 (0.65)	2.75 (0.11)	6.7 (6.33)	0.55 (1.88)	−2.25 ***	0.96
					1.70 n.s.	---
					−4.45 ***	3.67
					3.95 ***	1.68
					−2.20 ***	1.11
					−6.15 ***	4.54
**PIGDS sum score** **mean (SE)**	5.56 (0.59)	3.04 (0.11)	6.33 (0.42)	1.18 (0.11)	−2.52 ***	1.01
					0.76 n.s.	---
					−4.38 ***	2.36
					3.29 ***	1.3
					−1.86 ***	0.82
					−5.15 ***	2.57
**Gaming days per week** **mean (SE)**	5.88 (0.33)	5.54 (0.99)	5.93 (0.28)	4.42 (0.13)	−0.34 n.s.	---
					0.06 n.s.	---
					−1.45 p_uncorr_ = 0.04	0.68
					0.40 n.s.	---
					−1.12 ***	0.56
					−1.51 ***	0.71
**Gaming hours per day** **mean (SE)**	171.75 (41.91)	136.76 (5.03)	215.55 (25.27)	76.76 (3.84)	−34.99 n.s.	---
					43.80 n.s.	---
					−94.99 *	1.3
					78.80 ***	0.67
					−60.00 ***	0.62
					−138.79 ***	1.59
**DERS sum score** **mean (SE)**	49.69 (3.39)	41.88 (0.53)	54.89 (1.79)	34.02 (0.61)	−7.81 n.s.	---
					5.20 n.s.	---
					−15.67 ***	1.52
					13.01 ***	1.11
					−7.86 ***	0.7
					−20.87 ***	2
**Days of absence** **mean (SE)**	2.38 (0.75)	1.89 (0.19)	5.61 (1.19)	1.51 (0.18)	−0.48 n.s.	---
					3.23 n.s.	---
					−0.87 n.s.	---
					3.72 ***	0.8
					−0.38 n.s.	---
					−4.10 ***	1
**Grades sum mean (SE)**	6.06 (0.55)	6.37 (0.11)	6.70 (0.43)	6.06 (0.14)	---	---
**Grades development** **mean (SE)**	3.38 (0.18)	3.14 (0.03)	2.78 (0.11)	3.28 (0.04)	−0.23 n.s.	---
					−0.59 p_uncor r_ = 0.02	0.79
					−0.10 n.s.	0.14
					−0.36 *	0.56
					0.14 p_uncorr_ = 0.05	0.21
					0.50 ***	0.73

**Notes:** Bonferroni-corrected *p*-values: *** *p* ≤ 0.001, * *p* ≤ 0.05; p_uncorr_ = uncorrected *p*-Value; MANOVA = Multivariate Analysis of Variance, LPA = Latent Profile Analysis, x^2^ = chi-square, ^a^ post-hoc tests reported in the following sequence: IG-HG, PG-IG, LG-HG, PG-IG, LG-IG, LG-PG; Cramér’s V/Cohen’s d = effect sizes, [95%-CI] = 95% confidence interval, SE = standard error of the mean, days of absence in school/apprenticeship/job, grade sum = cumulated grades of the three main subjects with higher scores indicating poorer performance, grades development = improvement of grade sum over last term, GADIS-A = Gaming Disorder Scale for Adolescents, GADIS-A factor 1 = negative consequences, GADIS-A factor 2 = cognitive-behavioral symptoms, IGDS = Internet Gaming Disorder Scale, PIGDS = Parental Internet Gaming Disorder Scale, DERS = Difficulties in Emotion Regulation Scale.

**Table 6:** Post-hoc MANOVA and between groups tests in GD/ Non-GD adolescents according to ROC-cut offs and time criterion (GD symptoms during the past year at least for longer periods or daily).

Variables	No GD	GD	χ2/Scheffé	Cramer’s V/Cohen’s d
Absolute frequency	789	30	-	-
Relative frequency [95% CI]	96.34 [95.05, 97.62]	3.66 [2.38; 4.95]	-	-
Mean age (SE)	12.98 (0.08)	13.30 (0.36)	-	-
Female sex [95% CI]	40.68 [37.26, 44.11]	26.67 [10.84; 42.49]	-	-
GADIS-A factor 1 score mean (SE)	2.13 (0.11)	13.30 (0,78)	11.17 ***	3.57
GADIS-A factor 2 score mean (SE)	4.94 (0.13)	13.53 (0.39)	8.59 ***	2.34
Frequency score of GD symptoms mean (SE)	0.70 (0.02)	2.63 (0.09)	1.93 ***	3.31
IGDS sum score mean (SE)	2.08 (0.08)	7.47 (0.39)	5.39 ***	2.32
PIGDS sum score mean (SE)	2.50 (0.09)	6.47 (0.55)	3.97 ***	1.55
Gaming days per week mean (SE)	5.15 (0.07)	6.33 (0.29)	1.19 **	0.58
Gaming hours per day mean (SE)	117.7 (19.4)	225.52 (26.01)	107.82 ***	1.00
DERS sum score mean (SE)	39.47 (0.43)	57.00 (2.31)	17.53 ***	1.46
Days of absence mean (SE)	1.79 (0.13)	7.03 (1.72)	5.24 ***	1.28
Grades sum mean (SE)	6.26 (0.09)	6.83 (0.59)	-	-
Grades development mean (SE)	3.19 (0.02)	2.67 (0.12)	−0.52 ***	0.81

*p* values: *** *p* ≤ 0.001, ** *p* ≤ 0.01; MANOVA = Multivariate Analysis of Variance, GD = Gaming Disorder, ROC = Receiver Operating Characteristic, x^2^ = chi-square, Cramér’s V/Cohen’s d = effect sizes, [95%-CI] = 95% confidence interval, SE = standard error of the mean, absolute frequency of gaming, relative frequency of gaming, GADIS-A = Gaming Disorder Scale for Adolescents, GADIS-A factor 1 = negative consequences, GADIS-A factor 2 = cognitive-behavioral symptoms. IGDS = Internet Gaming Disorder Scale, PIGDS = Parental Internet Gaming Disorder Scale, DERS = Difficulties in Emotion Regulation Scale, days of absence from school/apprenticeship/job, grade sum = cumulated grades of the three main subjects with higher scores indicating poorer performance, grades development = improvement of grade sum over last term.

## Supplementary Materials

The following are available online at https://www.mdpi.com/2077-0383/9/4/993/s1, Table S1: German GADIS-A; Table S2: Inter-item correlations of GADIS-A.; Table S3: Relative item-response frequency of GADIS-A; Table S4: MANOVAs with GD and Non-GD as well as the four LPA profiles as dependent variables.
