# Supplementary material for: Assessing ICD-11 Gaming Disorder in Adolescent Gamers: Development and Validation of the Gaming Disorder Scale for Adolescents (GADIS-A)
Source: J Clin Med. 2020 Apr 2;9(4):993. doi: 10.3390/jcm9040993 (PMC7230491; doi:10.3390/jcm9040993)
Supplement: Supplementary file 1 [file jcm-09-00993-s001.pdf]

Supplementary Materials

Table S1. German GADIS-A.

**GADIS-A Fragebogen**	
**Wenn Du an die letzten 12 Monate denkst, wie stark stimmst Du mit den folgenden Aussagen überein?**	
1.	Ich spiele oft häufiger und länger, als ich es geplant oder mit meinen Eltern vereinbart habe.[1]
2.	Ich kann oft nicht aufhören zu spielen, obwohl es vernünftig wäre, dies zu tun, oder zum Beispiel meine Eltern mir gesagt haben, ich solle aufhören.[1]
3.	Ich verfolge oft keine Interessen außerhalb der digitalen Welt (z.B. Freunde oder Partner im wirklichen Leben treffen, Vereine besuchen, Bücher lesen, Musik machen), weil ich lieber spiele.[1]
4.	Ich vernachlässige meine täglichen Pflichten (z.B. Lebensmittel einkaufen, putzen, aufräumen, Verpflichtungen für Schule/Lehrstelle/Job), weil ich lieber spiele.[1]
5.	Normalerweise spiele ich weiter, auch wenn es mir Stress mit anderen (z.B. meinen Eltern, Geschwistern, Freunden, Partnern, Lehrern) bereitet.[1]
6.	Ich spiele weiter, obwohl es meine Leistungen in der Schule/ meine Lehrstelle/ meinen Job beeinträchtigt (z.B. durch Verspätung, Fehlzeiten, Vernachlässigung der Hausaufgaben, schlechtere Noten).[1]
7.	Durch das Spielen vernachlässige ich mein Aussehen, meine Hygiene und/oder meine Gesundheit (z.B. in Bezug auf Schlaf, Ernährung, Bewegung).[1]
8.	Durch das Spielen riskiere ich, wichtige Kontakte (Freunde, Familie, Partner) zu verlieren oder habe sie bereits verloren.[1]
9.	Durch das Spielen habe ich Nachteile in der Schule / der Lehre / im Beruf (z.B. schlechte (Abschluss-) Noten, Versetzungsgefährdung / kein Abschluss, keine Lehrstelle oder Studienplatz, schlechtes Zeugnis, Abmahnung / Entlassung).[1]
10.	Wie oft hattest Du im vergangenen Jahr mit solchen Problemen, Konflikten oder Schwierigkeiten aufgrund des Spielens zu tun? War das an einzelnen Tagen, über längere Zeiträume von mehreren Tagen bis Wochen oder fast täglich?[2]

Notes: [1] Antwortmöglichkeiten: GADIS-A = Gaming Disorder Scale for Adolescents; 5-Punkte-Likert-Skala: "stimme nicht zu" bis "stimme vollkommen zu"; [2] Antwortmöglichkeiten: "überhaupt nicht", "nur an einzelnen Tagen", "über längere Zeiträume", "fast täglich".

Table S2. Inter-item correlations of GADIS-A.

Item[a]	Item 1	Item 2	Item 3	Item 4	Item 5	Item 6	Item 7	Item 8	Item 9	Time Criterion
Item 1	-									
Item 2	0.74	-								
Item 3	0.45	0.49	-							
Item 4	0.51	0.54	0:58	-						
Item 5	0.63	0.67	0.59	0.64	-					
Item 6	0.44	0.47	0.59	0.63	0.60	-				
Item 7	0.33	0.37	0.58	0.46	0.45	0.59	-			
Item 8	0.38	0.41	0.62	0.46	0.51	0.59	0.67	-		
Item 9	0.36	0.40	0.58	0.53	0.50	0.76	0.67	0.70	-	
Time Criterion	0.68	0.66	0.50	0.54	0.61	0.46	0.42	0.44	0.45	-

Notes: N = 819, GADIS-A = Gaming Disorder Scale for Adolescents. Italicized figures indicate inter-item correlations within the subscale, [a]For the description of the items, refer to Table 1.

Table S3. Relative item-response frequency of GADIS-A.

Response Options / Item[a]	Strongly disagree	Somewhat disagree	Partially agree/ partially disagree	Somewhat agree	Strongly agree
Item 1	0.20	0.24	0.29	0.18	0.09
Item 2	0.27	0.25	0.24	0.16	0.08
Item 3	0.61	0.21	0.10	0.06	0.02
Item 4	0.43	0.28	0.18	0.09	0.03
Item 5	0.45	0.25	0.18	0.09	0.04
Item 6	0.62	0.23	0.08	0.05	0.02
Item 7	0.76	0.14	0.05	0.03	0.01
Item 8	0.74	0.19	0.04	0.02	0.02
Item 9	0.72	0.17	0.06	0.03	0.01
	**Not at all**	**Only on single days**	**For longer periods**	**Nearly daily**	
**Time Criterion**	0.34	0.59	0.04	0.04	

Notes: GADIS-A = Gaming Disorder Scale for Adolescents, [a]for the description of the items, refer to Table 1.

Table S4. MANOVAs with GD and Non-GD as well as the four LPA profiles as dependent variables.

	**GD and Non-GD**	**LPA profiles**
Variables	**F-value**	**F-value**
Age	0.23	0.98
Sex	1.66	9.49***
GADIS-A sum score factor 1	385.33***	114.89***
GADIS-A sum score factor 2	130.84***	412.61***
Frequency of GD symptoms	322.20***	316.89***
IGDS sum score	155.58***	157.91***
PIGDS sum score	68.78***	89.17***
Gaming days per week	9.37**	22.09***
Gaming hours per day	27.39***	34.67***
DERS sum	59.48***	55.54***
Days of absence	44.23***	11.52***
Grade sum	1.32	1.42
Grade development	16.19***	5.69***

Notes: *** $p \leq .001$, ** $p \leq .01$, * $p \leq .05$; MANOVA = Multivariate Analysis of Variance, GD = Gaming Disorder, LPA = Latent Profile Analysis, GADIS-A = Gaming Disorder Scale for Adolescents, GADIS-A factor 1 = negative consequences, GADIS-A factor 2 = cognitive-behavioral symptoms IGDS = Internet Gaming Disorder Scale, PIGDS = Parental Internet Gaming Disorder Scale, DERS = Difficulties in Emotion Regulation Scale, days of absence in school/ at work, Grade sum = cumulated grades of the three main subjects with higher scores indicating poorer performance, Grades development = improvement of grade sum during the past year.
